# Long-term effects of the titanium butterfly implant on quality of life: a prospective cohort study

**DOI:** 10.1007/s00405-024-08703-z

**Published:** 2024-05-03

**Authors:** F. S. Harthoorn, K. J. A. O. Ingels, G. W. J. A. Damen, A. R. T. Scheffer, N. van Heerbeek

**Affiliations:** 1 Faculty of Medical Sciences, Radboud University, Nijmegen, the Netherlands; 2 Department of Otorhinolaryngology, Radboud University Medical Center, Head and Neck (Number 383), Postbus 9109, 6500 HB Nijmegen, the Netherlands

**Keywords:** Nasal obstruction, Prostheses and implants, Quality of life, Rhinoplasty, Titanium

## Abstract

**Purpose:**

Nasal valve insufficiency is known to have a negative impact on both nasal patency and quality of life. The titanium butterfly implant is a surgical treatment proven to have a positive effect on these aspects up to 6 months postoperative. This study aimed to determine the long-term effects of the titanium butterfly implant on nasal obstruction symptoms and quality of life in adult patients with nasal valve insufficiency up to 5 years after procedure.

**Methods:**

A prospective single cohort study was performed including 29 patients that underwent the titanium butterfly implant in one tertiary medical center. Data was obtained before and at least 5 years after surgery using three questionnaires: the Nasal Obstruction and Septoplasty Effectiveness questionnaire, the Sino-Nasal Outcome Test 22 and the Glasgow Benefit Inventory questionnaire.

**Results:**

A significant decrease in total NOSE score was seen compared to baseline measurements. The SNOT-22 scores also showed a significant decrease, whereas the GBI scores showed no significant changes at the late follow-up.

**Conclusion:**

Seven years after placement the titanium butterfly implant still has a statistically significant improvement on otorhinologic-related quality of life compared to preoperative measurements.

## Introduction

**Fig. 1:**
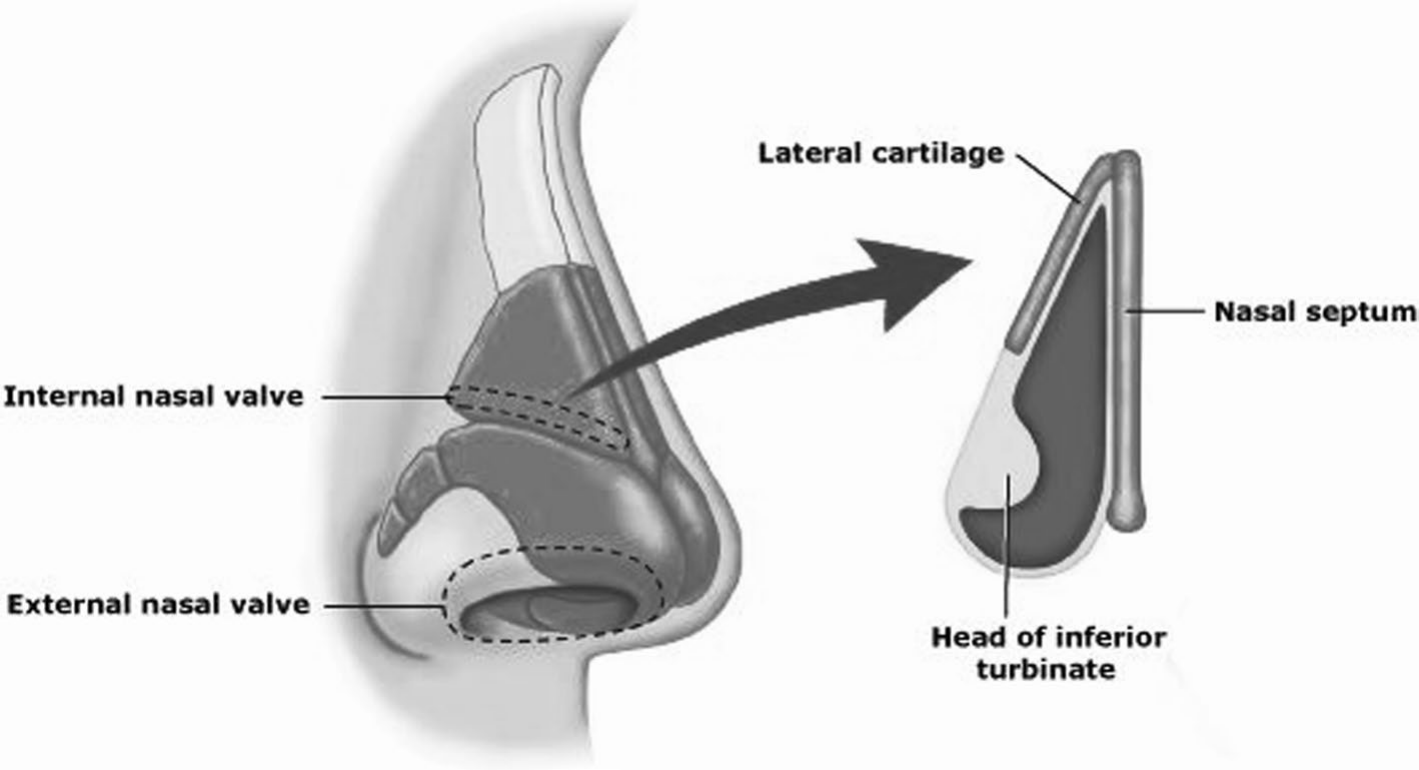
Anatomy of the Nasal Valves [20]

Nasal valve insufficiency, or nasal valve collapse, is known to have a negative impact on quality of life (QoL) due to nasal obstruction symptoms [1]. This can be explained from the anatomy of the nasal valve region, which consist of the internal and external nasal vale (INV and ENV). The INV lies cranial to the ENV and is the narrowest part of the nasal airway. It is formed by the caudal border of the upper lateral cartilage (ULC), the head of the inferior turbinate, the floor of the nasal cavity and the adjacent septum (Fig. 1). The ENV consist of the septum caudal to the INV, the alar cartilage and the soft tissue of the lower lateral wall. Both at the level of the INV as well as the ENV the lateral wall can move medially during breathing or even collapse.

This inward movement is influenced by strength of breathing and abnormalities in this region can easily result into nasal obstruction symptoms [2]. Underlying causes encompass numerous pathologies and physiologic age-related changes (i.e. loss of elastic properties and nasal muscle tone resulting in weakening of the lateral cartilaginous nasal wall and a drooping tip) [3], contributing to a higher estimated prevalence in the elderly (60%) compared to general population (13%) [4]. This is anticipated to increase due to global aging, resulting into a higher demand for treatment [5].

Many developed treatments for nasal valve insufficiency are described in literature. Sinkler and colleagues [6] conducted an in-depth literature review to outline these surgical methods, including grafts, implants and suture suspension techniques. This resulted in a guide for surgeons to select an appropriate technique for individual patients. However, no technique was found to be more superior than others.

One of these described treatments is the titanium butterfly implant, also known as the breathe-implant (Heinz Kurz GmbH, Dusslingen, Germany). Since its implementation in 2003 [7], literature on the effects of this intervention is sparse. Advantages are minimal cosmetic deformity and the possibility to alter the titanium plate after implantation, whereas a disadvantage is a possible high extrusion rate [5, 6]. A prospective single cohort study showed both significant and clinically relevant objective and subjective improvements in nasal patency and QoL 6 months after surgery. Measurement tools used in this study were peak nasal inspiratory flow (PNIF) and three QoL questionnaires (NOSE, SNOT-22, GBI) [8]. To date, no other studies have been published on the effect of this implant.

Thus, literature on the long-term postoperative effects of the titanium butterfly implant is lacking. Although a 90% satisfaction rate after 5 years has been stated by Wengen, the study’s methodology remains unclear [2]. It is important to objectively study the long-term outcomes since results may diminish over an extended period. Notably, a previous study on the porous polyethylene implant revealed a significant amount of complications within 10 months postoperative, a timeframe surpassing the previously studied 6 months [9]. As a follow-up from the previous study of Van den Broek and Van Heerbeek [8], this study aims to determine the long-term effects of the titanium butterfly implant on disease-related QoL and nasal obstruction symptoms. As a secondary objective, long-term outcomes will be compared with previous outcomes reported at the 6 weeks and 6 months follow-up.

## Materials and methods

### Study design

A prospective, single cohort, nonrandomized, single-centered study was performed. Participants were preoperatively included and followed at least 5 years after surgery. Pre- and post-operative measurements on nasal patency and QoL were performed to determine the effect of the titanium butterfly implant. Ethical approval was waived due to the non-invasive character of the study.

### Study population

All patients scheduled for the titanium butterfly implant as treatment for nasal obstruction due to INV insufficiency at one tertiary medical center from August 2014 to July 2017 were asked to participate. Patients were preoperatively included after giving informed consent. Patients were included if they suffered from INV insufficiency with a surgery indication that was established by symptoms, surgical history and clinical examination. All patients had a positive Cottle maneuver and used a nasal spreader prior to surgery with a positive effect on their nasal obstruction. No specific exclusion criteria were set up, therefore allowing inclusion of patients with septal deviation, ENV insufficiency (which is also an indication for surgical placement of the implant) and/or a history of nasal surgery. Additionally, daily use of nasal spray or concurrent surgery during the placement of the implant were allowed. These criteria therefore aligned with the surgical indications of the implant used in daily practice.

### Data collection and management

Patients’ characteristics and other relevant factors (size of implant, surgeon, daily use of nasal spray and prior or concurrent surgery) was obtained. The Nasal Obstruction and Septoplasty Effectiveness (NOSE) and Sino-Nasal Outcome Test 22 (SNOT-22) questionnaire were used to obtain information on nasal patency and quality of life before surgery and were repeated 6 weeks, 6 months and at least 5 years after surgery. The NOSE questionnaire was used as primary outcome. The Glasgow Benefit Inventory (GBI) questionnaire was added at all postoperative measurements [10]. These questionnaires are all internationally used tools to subjectively measure otorhinologic-related QoL and nasal obstruction symptoms and are proven reliable and validated [11–14]. As an objective measurement for nasal patency, peak nasal inspiratory flow (PNIF) measurements were performed preoperatively and at 6 weeks and 6 months post-operatively. The PNIF was not performed at the late follow-up due to practical reasons.

### Surgical technique

**Fig. 2:**
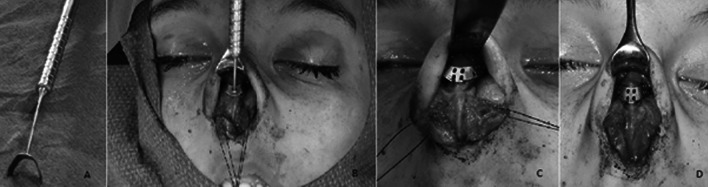
Information about the surgical procedure [8]. **a** Sizer-instrument. **b** Placing instrument to measure size at the cartilage nasal dorsum. **c** Placement of implant and fixation to triangular cartilages. **d** Repositioning of alar cartilages and final fixation of implant

All study participants were operated under general anesthesia between August 2014 and July 2017. Three ENT surgeons, all specialized in nasal surgery, performed the surgery. Some subjects had concurrent surgery. An open rhinoplasty approach was used in all patients. After exposure of the alar cartilage and cartilaginous nasal dorsum, sizer instruments were used to determine the width of the cartilaginous dorsum (Fig. 2). The chosen size of the implant was one to two sizes wider than the measured width and was documented in the medical file of the patient. With several Prolene 5–0 sutures, the graft was secured onto the cartilaginous nasal dorsum and upper lateral cartilages on both sides. The upper lateral cartilages were sutured outward towards the implant, thus widening the INV. Thereafter, the cephalic part of the lateral crus of the lower lateral cartilage was placed over the graft in order to support the ENV. Skin incisions were closed using a Monocryl 6–0 suture. During surgery all participants received prophylactic cefazolin followed by 1 week of amoxicillin-clavulanic acid postoperatively.

### Statistical analysis

Data were registered in IBM SPSS Statistics (IBM Corp. Released 2022. IBM SPSS Statistics for Windows, Version 29.0. Armonk, NY: IBM Corp). Descriptive statistics were used to summarize baseline characteristics and GBI measurements. Normality of data was determined using the Shapiro–Wilk test. Within subject analyses was done with paired t-tests to provide an estimated difference in mean and, when data were normally distributed, statistical difference was determined. The non-parametric Wilcoxon signed-rank or sign tests were used to determine statistical significance when non normality was found. Independent T-Tests, or Mann–Whitney U Tests if data were not normally distributed, were performed to enable analyses between two different groups. Confounding analysis was performed via one-way ANOVA. A p-value less than 0.05 was considered significant.

## Results

From the 29 patients initially included in the preoperative phase of the original study, 27 were approached for participation in the subsequent late follow-up. Contact information for the remaining two patients could not be ascertained. One participant did not complete the questionnaires since they had the implant removed due to no subjective effect and corresponding discomfort. Two other patients got excluded due to death unrelated to the surgery. A total of 22 patients (75.86%) replied with a mean follow-up time of 7.09 years (range 5.17–8.67 years, median = 7.2 years). Of those, two participants partially responded by only completing the NOSE questionnaire.

Baseline characteristics of the original study population are displayed in Table 1. Ratio men to women was approximately three to one with the mean age of all participants being 46.2 ± 11.5 years (median: 48.0). Prior surgery was performed in 25 participants and varied in frequency and type (from turbinate reduction to open rhinoplasty). Mean preoperative total scores were 75.34 points for the NOSE and 44.21 for the SNOT-22 questionnaire.

**Table 1:** Baseline characteristics of the original study population

	*n*	*%*	Mean	Maximum	Minimum	Standard deviation
Gender						
Male	21	72.4				
Female	8	27.6				
Age at time of operation	29		46.2	65	22	11.5
Prior surgery						
No	4	13.8				
Yes	25	86.2				
Use of daily nasal spray						
No	28	96.6				
Yes	1	3.4				
Baseline NOSE total score	29		75.3	100.0	40.0	16.3
Baseline SNOT-22 total score	29		44.2	87.0	18.0	19.4
Surgeon						
A	2	6.9				
B	26	89.7				
C	1	3.4				
Size of implant						
L	8	27.6				
XL	13	44.8				
XXL	8	27.6				
Concurrent surgery						
No	22	75.9				
Yes	7	24.1				

**Fig. 3:**
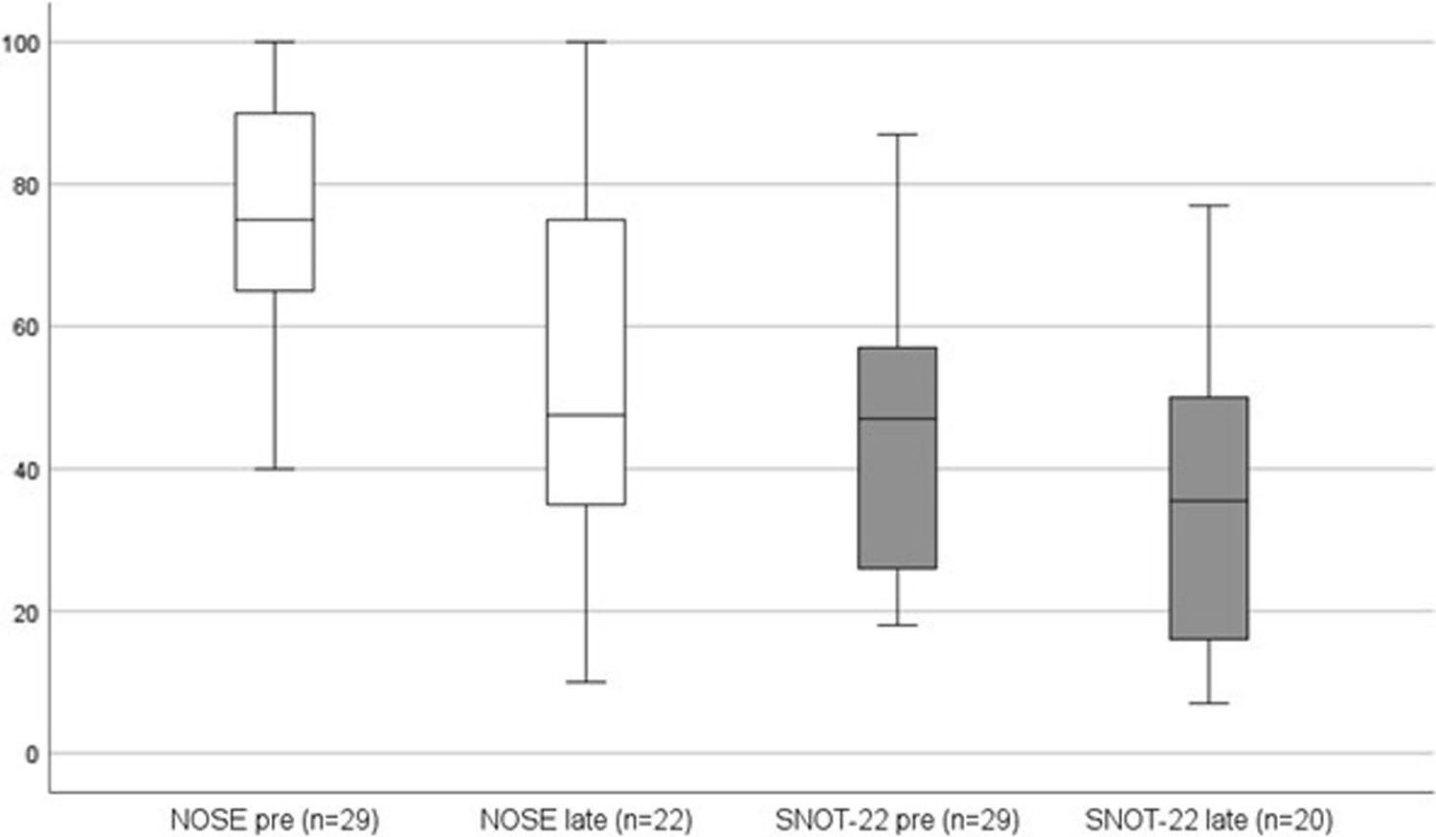
Mean total scores of the NOSE and SNOT-22 questionnaires at preoperative measurements and the late follow-up. A higher score means more experienced symptoms and a negative impact on quality of life. *NOSE* Nasal Obstruction and Septoplasty Effectiveness questionnaire, *SNOT* Sino-Nasal Outcome Test 22, *n* amount of responders at the specific follow-up, *Pre* preoperative measurements, *Late* late follow-up, with a mean of 7 years after surgery

The mean total NOSE score of 22 participants was 54.32 points at the late follow-up and showed a significant decrease of 22.27 points (95% CI 11.64–32.91, p < 0.001) compared to baseline measurements, as shown in Fig. 3. Compared to the NOSE score at 6 weeks and 6 months the late NOSE score, though still being lower than before surgery, was higher with a difference of respectively 14.06 (n = 16) and 27.00 (n = 15) points. Regarding these distinctions, the mid- to long-term outcomes significantly differed (p = 0.002).

The mean total SNOT-22 score was 34.80, which was 10.00 points lower compared to baseline outcomes (n = 20) (Fig. 3). The difference was proven significant (p = 0.019). Like with the NOSE, the late SNOT-22 total score was higher compared to both short- and mid-term scores; respectively 7.36 points (n = 14) and 16.92 points (n = 13) within subjects. This last difference was proven statistically significant (p = 0.010).

At the late follow-up (n = 20), both total as well as subsets scores on the GBI questionnaire showed no significant postinterventional change. This is in contrast with the GBI results at 6 weeks and 6 months, that both showed significant positive total scores. An overview of the mean scores at all follow-up moments is displayed in Table 2. Scores recorded at 6 weeks and 6 months were consistently higher than those observed during the late follow-up. Further within-subject analyses revealed that solely the total scores at 6 weeks, the general subset scores at 6 weeks and the physical health subset scores at 6 months significantly differed compared to late outcomes.

**Table 2:** An overview of the mean scores on the GBI questionnaire at all follow-ups, including standard deviation (SD) and 95% Confidence Interval (CI)

	6 weeks (n = 20)	6 months (n = 18)	Late (n = 20)
Total *Mean* ± *SD, [95% CI]*	11.53 ± 11.37 [6.2087, 16.8513]*	20.22 ± 24.53 [8.0215, 32.4185]*	4.17 ± 22.38 *[− 6.3042, 14.6442]*
General *Mean* ± *SD, [95% CI]*	14.38 ± 14.01 [7.8231, 20.9369]*	22.92 ± 26.01 [9.9855, 35.8545]*	5.42 ± 27.58 *[− 7.4878, 18.3278]*
Social support *Mean* ± *SD, [95% CI]*	8.33 ± 15.77 [0.9494, 15.7106]*	5.56 ± 23.57 *[− 6.1611, 17.2811]*	0.00 ± 10.81 *[− 5.0592, 5.0592]*
Physical health *Mean* ± *SD, [95% CI]*	3.33 ± 10.26 *[− 1.4718, 8.1318]*	24.07 ± 35.34 [6.4958, 41.6442]*	3.33 ± 28.92 *[− 10.205, 16.865]*

The test in italics is the 95% Confidence Interval

*n* = amount of responders at specific follow-up

^*^Statistically significant difference

Three patients had revision surgery after placement of the titanium butterfly implant (10.3%). One implant was removed 25 months after implantation because the patient experienced no positive effect. One implant was repositioned after shifting from its origin, 22 months after initial placement. The last patient received additional septal and bilateral inferior turbinate reduction after 22 months with subjective positive outcomes on nasal patency. No other complications, such as postoperative infections or extrusion, were reported.

A characteristic of the implant is that it can be bend relatively easily. Therefore, the implant can be deformed over time and the nasal valve region can consequently become smaller again. It is possible to re-bend the implant at the outpatient clinic. For each participants, the frequency of this bending (range 0–4) and subjectively corresponding effect was documented. No questionnaires were used to objectify the immediate effect. Half of the participants (n = 15, 51.7%) had the implant bend at least once. The majority of these patients (86.7%) reported an immediate subjective improvement of the nasal patency. Seven patients had the implant bend twice (24.1%), four patients thrice (13.8%) and two patients four times (6.9%). The appropriate degree of bending was determined using directly subjective outcomes.

Concurrent surgery (n = 5), including septoplasty (n = 3), reconstruction of the lateral crus (n = 1) and reduction of the cartilage hump with reduction of pre-existing posterior graft (n = 1), showed no significant difference (F(2) = 2.578, p = 0.124) on NOSE total score at the late follow-up compared to participants that solely had the implant placed (n = 17).

Seven patients from the original study population (n = 29) did not participate in this follow-up study. Previously reported outcomes, specifically the difference in NOSE total score between baseline and 6 months measurements, of participants and non-responders at the late follow-up were compared with each other to rule out selection bias. No significant difference was found (p = 0.301).

## Discussion

This study aimed to determine the long-term effects of the titanium butterfly implant on nasal obstruction symptoms and corresponding QoL. The results, based on validated and reliable questionnaires [11–14], demonstrated a significant improvement of disease-related QoL persisting for an average of 7 years after placement of the titanium butterfly implant. No long term complications, such as extrusion, were found.

The late NOSE total score showed a significant decrease of 22.3 points on a scale of hundred. Since the NOSE total score can be divided into clinically subcategories of severity that all consist of 20 points [15], this also implies a mean decrease of one level in clinical severity. We therefore consider this difference clinically relevant, even though previous literature has only described 30 points to be the minimal clinical important difference (MCID) [16]. Care should be noted that this difference has been based on studies with an average follow-up of 2 months or more. The follow-up of this study is considerably longer and should be taken into account while discussing which difference in total score is clinically relevant.

The SNOT-22 total score also showed a significant decrease at the late follow-up compared to preoperative measurements. This drop of ten points meets the commonly used MCID of 8.9 points proven by Hopkins and colleagues [12] and is therefore considered clinically relevant.

The GBI questionnaire showed no significant difference in QoL due to the intervention. This discrepancy with the other results may be attributed to the broader nature of the questions incorporated in the GBI. Over the 7-year period, it is speculated that these questions could be susceptible to other factors and recall bias. While a study in 2008 found persistent positive scores on the GBI questionnaire when comparing 5–8 years (with an average of 6 years) postoperative results with short-term (3 months) outcomes [17], another study on septal surgery did not identify significant changes using the GBI questionnaire up to 3 years postoperative [18].

The results further revealed the late outcomes to be less distinct than the short- to mid-term results on all questionnaires. These changes suggest a diminishing effect over time and deserve more attention due to their extent and clinical relevance. It is debatable whether this diminishing effect is only due to the commonly mentioned postoperative “placebo” effect or if response bias, where patients may be inclined to provide a positive review shortly after surgery to please the doctor, and other factors influence the effect of the implant over an extended period [18].

We speculate that the easy-bend characteristic of the implant and the influence of aging on both nasal obstruction symptoms and experienced quality of life could affect the long-term outcomes. Patients could have altered the shape of the implant by exerting external pressure (e.g., during periods of frequent sneezing, nose blowing or due to new nasal trauma). Nonetheless, this characteristic also offers advantages by allowing reshaping of the implant in an outpatient clinic resulting into a wider nasal valve region and a subjective improvement of nasal patency. Aging is known to result into nasal symptoms, such as rhinorrhea and an increased incidence of nasal polyposis, and could therefore have affected the outcomes on all three questionnaires [19].

Strengths of this study are the long follow-up, the prospective study-design, and the use of validated and reliable questionnaires. Therefore, subjective experienced symptoms could be objectified and compared within subjects over time. By using the NOSE and SNOT-22 questionnaires at specific moments over time, the authors believe there is limited recall bias. These questionnaires are based on the currently experienced symptoms. Nevertheless, this study is subject to some limitations. Firstly, the sample size was small. Therefore, confounding analysis was limited and the power could be compromised. Secondly, it would have been an added value to be able to compare the subjective outcomes with objective values using for example peak nasal inspiratory flow (PNIF). This was not incorporated in the late follow-up of the study due to practical reasons. Nevertheless, a negative correlation has been proved between PNIF and subjective questionnaires which is explained by disease-related quality of life being a different construct than nasal patency [13]. Since the aim of this study is an exploration of quality of life, the necessity of an objective measurement tool is therefore debatable. Lastly, there was no control group in this study. By incorporating a control group into the study design it would have been possible to compare results with either another treatment or no treatment; which in both cases could also facilitate analysis of confounding due to aging. Nonetheless, this is the first study showing the long term effectiveness of the titanium butterfly implant.

In summary, this study proved the titanium butterfly implant still being effective on QoL and nasal obstruction symptoms 7 years after surgery. Although the effect diminishes somehow over the years, the effect is still significant and clinically relevant.

## Data Availability

The datasets generated and analyzed during the current study are not publicly available since individual privacy could potentially be compromised. However, the datasets are available from the corresponding author on reasonable request.
